# Supplementary material for: Determining Perceived Self-Efficacy for Preventing Dengue Fever in Two Climatically Diverse Mexican States: A Cross-Sectional Study
Source: Behav Sci (Basel). 2022 Mar 28;12(4):94. doi: 10.3390/bs12040094 (PMC9031455; doi:10.3390/bs12040094)
Supplement: Supplementary file 1 [file behavsci-12-00094-s001.zip › MS.hs_behavior_supplementary tables_final.pdf]

Table S1: Knowledge about how dengue fever is transmitted statistics for different socio-economic parameters, by state.

State	Variable		Mean	Count	Std	95% CI_U	95% CI_L
Colima	Gender	Male	1.30	69	0.57	1.14	1.46
		Female	1.15	91	0.43	1.06	1.25
	Residence Area	Rural	1.17	7	0.41	0.74	1.60
		Urban	1.21	153	0.50	1.13	1.30
	Kind of Home	Single-family home	1.23	116	0.54	1.12	1.34
		*Others	1.16	44	0.37	1.04	1.28
Sonora	Gender	Male	1.36	176	0.65	1.25	1.48
		Female	1.27	346	0.52	1.21	1.33
	Residence Area	Rural	1.20	49	0.46	1.05	1.34
		Urban	1.31	473	0.58	1.25	1.37
	Kind of Home	Single-family home	1.29	392	0.55	1.23	1.36
		*Others	1.33	130	0.61	1.21	1.45
Other States	Gender	Male	1.33	40	0.48	1.15	1.51
		Female	1.24	73	0.60	1.08	1.39
	Residence Area	Rural	1.00	6	0	-	-
		Urban	1.29	107	0.57	1.16	1.41
	Kind of Home	Single-family home	1.25	65	0.56	1.10	1.40
		*Others	1.30	48	0.57	1.11	1.49

Table S2.1: Knowledge about symptoms associated with dengue fever statistics for different socio-economic parameters by state.

State	Variable		Mean	Count	Std	95% CI_U	95% CI_L
Colima	Gender	Male	5.87	311	2.24	5.25	6.49
		Female	5.66	447	1.95	5.22	6.09
	Residence Area	Rural	4.33	26	1.86	2.38	6.29
		Urban	5.81	732	2.06	5.45	6.17
	Kind of Home	Flats/Apartment	4.50	9	2.12	-14.56	23.56
		Semi-detached	6.29	107	2.26	5.13	7.45
		Single family home	5.79	544	2.06	5.36	6.21
		Townhouse	5.11	92	1.90	4.16	6.06
		*Others	6.00	6	-	-	-
Sonora	Gender	Male	5.73	739	1.85	5.41	6.05
		Female	5.18	1410	1.77	4.97	5.40
	Residence Area	Rural	4.78	196	1.86	4.19	5.37
		Urban	5.43	1953	1.80	5.24	5.61
	Kind of Home	Flats/Apartment	4.74	90	2.16	3.70	5.78
		Semi-detached	5.32	181	1.84	4.68	5.96
		Single family home	5.40	1637	1.80	5.20	5.61
		Townhouse	5.61	202	1.68	5.04	6.18
		*Others	4.33	39	1.80	2.95	5.72
Other States	Gender	Male	5.73	172	1.48	5.18	6.29
		Female	5.51	325	1.99	4.99	6.03
	Residence Area	Rural	4.00	24	2.00	1.90	6.10
		Urban	5.70	473	1.78	5.31	6.09
	Kind of Home	Flats/Apartment	4.44	40	1.13	3.58	5.31
		Semi-detached	6.75	81	2.01	5.48	8.02
		Single family home	5.71	297	1.83	5.20	6.22
		Townhouse	5.25	63	1.22	4.48	6.02
		*Others	4.00	16	2.16	0.56	7.44

Table S2.2: Knowledge about how dengue fever is transmitted statistics for different socio-economic parameters.

**Variable**		**Mean**	**Count**	**Std**	**95% CI_U**	**95% CI_L**
Gender	Male	1.34	285	0.61	1.26	1.43
	Female	1.24	510	0.52	1.19	1.29
Residence Area	Rural	1.17	62	0.43	1.05	1.29
	Urban	1.29	733	0.56	1.24	1.33
Kind of Home	Flats/Apartment	1.33	40	0.61	1.11	1.56
	Semi-detached	1.25	79	0.57	1.11	1.40
	Single family home	1.28	573	0.55	1.23	1.33
	Townhouse	1.32	87	0.56	1.18	1.46
	*Others	1.14	16	0.36	0.93	1.35
State	Colima	1.21	160	0.49	1.13	1.30
	Sonora	1.30	522	0.57	1.25	1.36
	ᵻOther states	1.27	113	0.56	1.15	1.39

*Others includes structures like Terrace, Duplex and Condominium.

ᵻOther states include states like Morelos, Nuevo Leon, Baja California, Chiapas, Ciudad de Mexico, Durango, Jalisco, Queretaro, Puebla, San Luis Potosi, Guerrero and Zacatecas.

Table S2.3: Knowledge about how to reduce dengue transmission statistics for different socio-economic parameters.

Variable		Mean	Count	Std	95% CI_U	95% CI_L
Gender	Male	4.56	967	2.19	4.27	4.86
	Female	4.34	1779	2.09	4.14	4.54
Residence Area	Rural	3.75	199	2.21	3.15	4.36
	Urban	4.48	2547	2.11	4.30	4.65
Kind of Home	Flats/Apartment	4.37	131	1.97	3.63	5.10
	Semi-detached	4.84	305	1.71	4.41	5.27
	Single family home	4.34	1949	2.20	4.14	5.27
	Townhouse	4.70	310	2.00	4.21	5.19
	*Others	3.64	51	2.10	2.43	4.85
State	Colima	4.58	604	2.33	4.18	4.98
	Sonora	4.30	1726	2.06	4.10	4.51
	ᵻOther states	4.67	416	2.06	4.24	5.11

*Others includes structures like Terrace, Duplex and Condominium.

ᵻOther states include states like Morelos, Nuevo Leon, Baja California, Chiapas, Ciudad de Mexico, Durango, Jalisco, Queretaro, Puebla, San Luis Potosi, Guerrero and Zacatecas.

Table S2.4: Knowledge about symptoms associated with dengue fever statistics for different socio-economic parameters.

Variable		Mean	Count	Std	95% CI_U	95% CI_L
Gender	Male	5.76	1222	1.91	5.51	6.02
	Female	5.32	2182	1.85	5.14	5.50
Residence Area	Rural	4.64	246	1.86	4.13	5.15
	Urban	5.55	3158	1.86	5.40	5.70
Kind of Home	Flats/Apartment	4.63	139	1.85	3.94	5.32
	Semi-detached	5.86	369	2.05	5.34	6.37
	Single family home	5.52	2478	1.86	5.35	5.69
	Townhouse	5.41	357	1.66	5.00	5.82
	*Others	4.36	61	1.82	3.30	5.41
State	Colima	5.74	758	2.07	5.39	6.10
	Sonora	5.36	2149	1.82	5.18	5.54
	ⱡOther states	5.59	497	1.83	5.20	5.97

*Others includes structures like Terrace, Duplex and Condominium.

ⱡOther states include states like Morelos, Nuevo Leon, Baja California, Chiapas, Ciudad de Mexico, Durango, Jalisco, Queretaro, Puebla, San Luis Potosi, Guerrero, and Zacatecas.

Table S2.5: Knowledge about climatic factors that affect dengue transmission statistics for different socio-economic parameters.

Variable		Mean	Count	Std	95% CI_U	95% CI_L
Gender	Male	2.40	506	1.46	2.20	2.60
	Female	2.08	842	1.45	1.94	2.23
Residence Area	Rural	2.08	110	1.25	1.73	2.42
	Urban	2.20	1238	1.48	2.08	2.33
Kind of Home	Flats/Apartment	2.23	67	1.38	1.72	2.75
	Semi-detached	2.03	128	1.48	1.66	2.40
	Single family home	2.21	977	1.45	2.07	2.35
	Townhouse	2.24	148	1.55	1.86	2.62
	*Others	2.00	28	1.57	1.09	2.91
State	Colima	2.17	287	1.44	1.93	2.42
	Sonora	2.16	853	1.49	2.02	2.31
	ⱡOther states	2.34	208	1.36	2.05	2.62

*Others includes structures like Terrace, Duplex and Condominium.

ⱡOther states include states like Morelos, Nuevo Leon, Baja California, Chiapas, Ciudad de Mexico, Durango, Jalisco, Queretaro, Puebla, San Luis Potosi, Guerrero and Zacatecas.

Table S3: Knowledge about how to reduce dengue transmission statistics for different socio-economic parameters by state.

State	Variable		Mean	Count	Std	95% CI_U	95% CI_L
Colima	Gender	Male	4.49	238	2.38	3.83	5.15
		Female	4.63	366	2.30	4.12	5.15
	Residence Area	Rural	4.33	26	2.34	1.88	6.79
		Urban	4.59	578	2.33	4.18	5.00
	Kind of Home	Flats/Apartment	6.00	12	1.41	-6.71	18.71
		Semi-detached	5.47	93	1.81	4.54	6.40
		Single family home	4.39	413	2.39	3.90	4.88
		Townhouse	4.61	83	2.40	3.42	5.81
		*Others	3.00	3	-	-	-
Sonora	Gender	Male	4.53	584	2.14	4.15	4.90
		Female	4.20	1142	2.02	3.96	4.44
	Residence Area	Rural	3.95	162	2.22	3.25	4.65
		Urban	4.34	1564	2.04	4.13	4.56
	Kind of Home	Flats/Apartment	4.32	82	2.16	3.27	5.36
		Semi-detached	4.32	147	1.57	3.78	4.87
		Single family home	4.28	1298	2.13	4.04	4.52
		Townhouse	4.58	165	1.83	3.97	5.20
		*Others	3.78	34	2.33	1.98	5.57
Other States	Gender	Male	4.83	145	2.07	4.06	5.61
		Female	4.59	271	2.07	4.05	5.13
	Residence Area	Rural	1.83	11	0.75	1.04	2.62
		Urban	4.88	405	1.97	4.45	5.31
	Kind of Home	Flats/Apartment	4.11	37	1.62	2.87	5.35
		Semi-detached	5.52	65	1.56	4.42	6.41
		Single family home	4.58	238	2.23	3.96	5.20
		Townhouse	5.17	62	1.95	3.93	6.40
		*Others	3.50	14	2.08	0.19	6.81

Table S4: Knowledge, Attitude, and Practice about Climate change and dengue fever

Variable		P-Value		
		Gender	Residence Area	State
Knowledge	Heard about Climate change	0.1436	0.8834	0.6339
	Perception that climate change is occurring	0.3794	0.0116	0.3247
	The perception that climate change affects the transmission of DF	0.0828	0.5497	0.2018
Attitude	Need for education programs on mitigation strategies related to climate change	0.0446	0.2876	0.0199
	Need for education programs on early warning signs related to climate change	0.3093	0.3228	0.0939
	Need for increased knowledge of how climate change can affect dengue fever	0.0275	0.1741	0.8588
	Need to reduce the risk of dengue caused by climate change	0.1982	0.7079	0.1391
	Need for government to take action to mitigate dengue fever risk that may be because of climate change	0.1934	0.2171	0.0081
Practice	Measures were taken to eliminate mosquitos in respondent's household in the last year.	0.9034	0.9125	0.1166
	Sprayed interior wall in the past 12 months	0.9695	0.9312	0.4061
	Used mosquito control against larvae in household	0.6926	0.1227	0.0368
	Used adult mosquito control in household	0.9790	0.6111	0.3996

Table S5.1: Measures taken by respondents in the previous year to control mosquitoes statistics for different socio-economic parameters.

Variable		Mean	Count	Std	95% CI_U	95% CI_L
Gender	Male	9.54	763	2.13	9.06	10.01
	Female	9.84	1269	2.22	9.45	10.22
Residence Area	Rural	8.79	167	3.21	7.24	10.34
	Urban	9.82	1865	2.05	9.52	10.11
Kind of Home	Flats/Apartment	9.18	101	2.67	7.38	10.98
	Semi-detached	10.00	270	2.25	9.11	10.89
	Single family home	9.73	1450	2.06	9.40	10.06
	Townhouse	9.63	183	2.89	8.24	11.02
	*Others	9.33	28	2.08	4.16	14.50
State	Colima	10.13	537	1.83	9.63	10.64
	Sonora	9.58	1264	2.32	9.18	9.97
	ⱡOther states	9.63	231	2.12	8.73	10.52

*Others includes structures like Terrace, Duplex and Condominium.

ⱡOther states include states like Morelos, Nuevo Leon, Baja California, Chiapas, Ciudad de Mexico, Durango, Jalisco, Queretaro, Puebla, San Luis Potosi, Guerrero and Zacatecas.

Table S5.2: Presence of items in respondent's yard statistics for different socio-economic parameters.

Variable		Mean	Count	Std	95% CI_U	95% CI_L
Gender	Male	2.39	481	1.60	2.17	2.62
	Female	2.24	844	1.44	2.09	2.38
Residence Area	Rural	2.45	125	1.29	2.09	2.81
	Urban	2.28	1200	1.52	2.15	2.41
Kind of Home	Flats/Apartment	2.21	62	1.03	1.81	2.61
	Semi-detached	2.12	127	1.37	1.76	2.47
	Single family home	2.32	965	1.51	2.17	2.47
	Townhouse	2.26	138	1.73	1.82	2.71
	*Others	2.53	33	1.33	1.73	3.34
State	Colima	1.82	227	1.36	1.57	2.06
	Sonora	2.47	905	1.52	2.31	2.62
	ᵻOther states	2.24	193	1.45	1.93	2.56

*Others includes structures like Terrace, Duplex and Condominium.

ᵻOther states include states like Morelos, Nuevo Leon, Baja California, Chiapas, Ciudad de Mexico, Durango, Jalisco, Queretaro, Puebla, San Luis Potosi, Guerrero and Zacatecas.

Table S5.3: The frequency of using the measure to control adult mosquitos statistics for different socio-economic parameters.

Variable		Mean	Count	Std	95% CI_U	95% CI_L
Gender	Male	1.81	383	1.44	1.61	2.00
	Female	1.91	783	1.54	1.76	2.06
Residence Area	Rural	1.79	95	1.56	1.36	2.22
	Urban	1.88	1071	1.50	1.76	2.01
Kind of Home	Flats/Apartment	2.10	63	1.67	1.48	2.73
	Semi-detached	2.21	139	1.59	1.81	2.61
	Single family home	1.85	829	1.47	1.71	1.98
	Townhouse	1.68	111	1.59	1.29	2.07
	*Others	1.71	24	1.49	0.85	2.57
State	Colima	1.68	222	1.37	1.45	1.92
	Sonora	1.86	746	1.54	1.71	2.01
	ᵻOther states	2.22	198	1.51	1.91	2.54

*Others includes structures like Terrace, Duplex and Condominium.

ᵻOther states include states like Morelos, Nuevo Leon, Baja California, Chiapas, Ciudad de Mexico, Durango, Jalisco, Queretaro, Puebla, San Luis Potosi, Guerrero and Zacatecas.

Table S6: Level of self-efficacy toward dengue prevention practices statistics for different socio-economic parameters.

Variable		Mean	Count	Std	95% CI_U	95% CI_L
Gender	Male	118.54	24894	20.03	115.82	121.27
	Female	117.70	48021	17.86	115.96	119.44
Residence Area	Rural	114.51	6069	17.09	109.80	119.22
	Urban	118.31	66846	18.73	116.76	119.86
Kind of Home	Flats/Apartment	110.50	3315	19.08	103.37	117.62
	Semi-detached	118.93	7255	18.23	114.26	123.60
	Single family home	118.16	52817	18.84	116.41	119.91
	Townhouse	118.75	7838	17.55	114.44	123.07
	*Others	120.71	1690	15.21	111.93	129.50
State	Colima	120.91	15598	16.15	118.10	123.73
	Sonora	116.99	46797	19.37	115.09	118.90
	ᵻOther states	118.20	10520	18.20	114.37	122.04

*Others includes structures like Terrace, Duplex and Condominium.

ᵻOther states include states like Morelos, Nuevo Leon, Baja California, Chiapas, Ciudad de Mexico, Durango, Jalisco, Queretaro, Puebla, San Luis Potosi, Guerrero and Zacatecas.

Table S7: Univariate and multivariate multiple linear regression analysis with mosquito control measures as a dependent variable.

**Independent Variables**	**Univariate**		**Multivariate**		
	β	p-value	β (Estimate)	Standardized estimate	p-value
Items in yard	-0.302	0.0007	-0.210	-0.167	0.0133
Age	0.007	0.5059	0.009	0.069	0.2996
Self-efficacy	0.045	<.0001	0.038	0.323	<.0001
Knowledge about dengue reduction practices	0.267	<.0001	0.246	0.316	<.0001

Table S8a: The results of multiple logistic regression analysis on predicting the odds of taking measures against mosquitoes the previous year.

Independent Variables	β	S. E	Significance	Prevalence OR	95% CI
Constant	-2.260	0.818	0.0057	-	-
Gender					
Female	0.019	0.139	0.8890	1.040	0.603 - 1.792
Male	0.000	-	-	1	-
Age	0.062	0.013	<.0001	1.064	1.036 - 1.092
Self-efficacy	0.020	0.007	0.0024	1.020	1.007 - 1.033

Table S8b: Univariate and multivariate multiple logistic regression analysis on predicting the odds of taking measures against mosquitoes the previous year.

Independent Variables	Univariate			Multivariate		
	OR	p-value	95% CI	AOR	p-value	95% CI
Gender						
Female	1.092	0.7404	0.648 - 1.840	1.040	0.8890	0.603 - 1.792
Male						
Age	1.067	<.0001	1.040 - 1.096	1.064	<.0001	1.036 - 1.092
Self-efficacy	1.022	0.0004	1.010 - 1.034	1.020	0.0024	1.007 - 1.033
