# Supplementary material for: Determining Perceived Self-Efficacy for Preventing Dengue Fever in Two Climatically Diverse Mexican States: A Cross-Sectional Study
Source: Behav Sci (Basel). 2022 Mar 28;12(4):94. doi: 10.3390/bs12040094 (PMC9031455; doi:10.3390/bs12040094)
Supplement: Supplementary file 1 [file behavsci-12-00094-s001.zip › MS_hs_behavior_supplementary_figures.pdf]

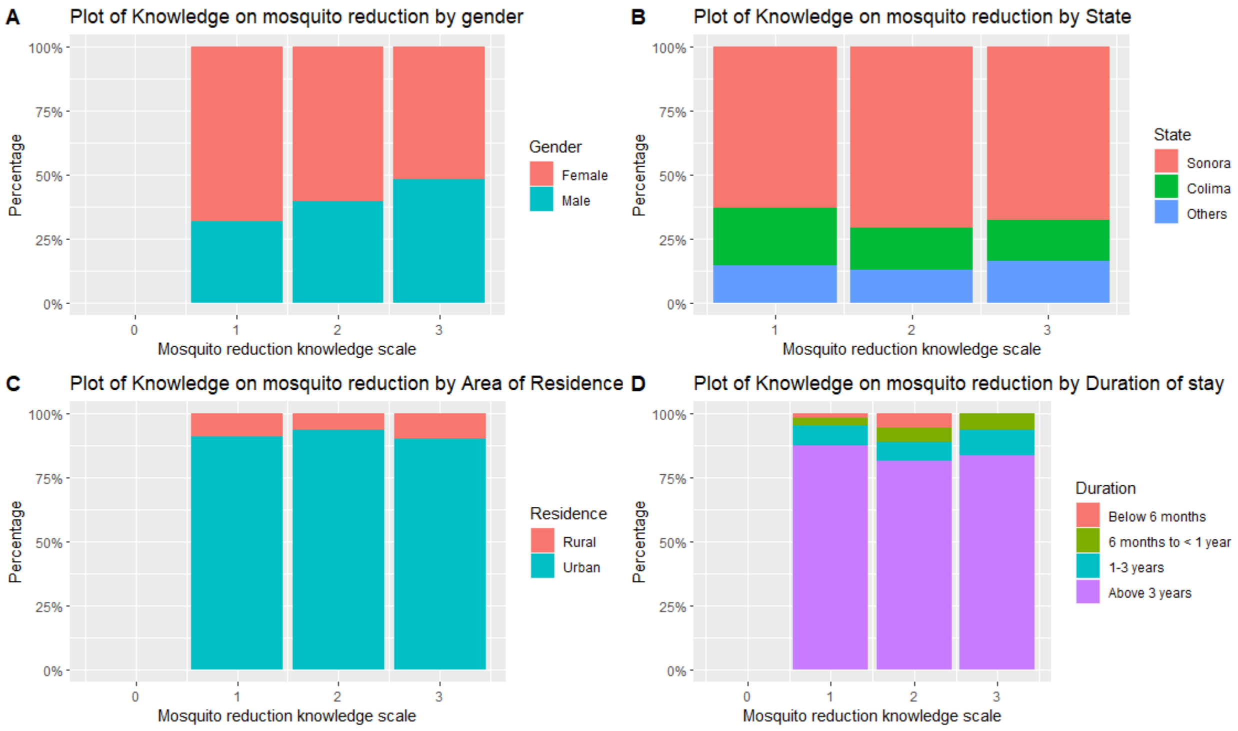

Figure S1. Distribution of knowledge on mosquito reduction by demographic factors.

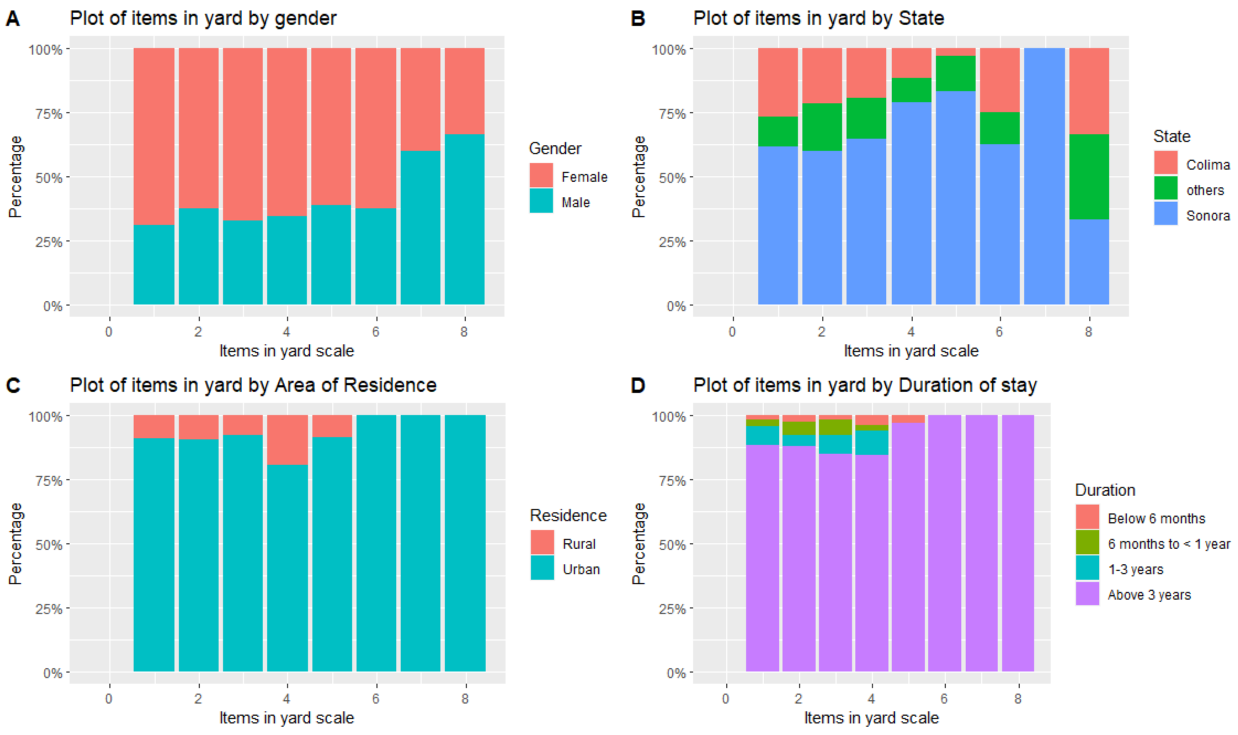

Figure S2. Distribution of items in yard by demographic factors.

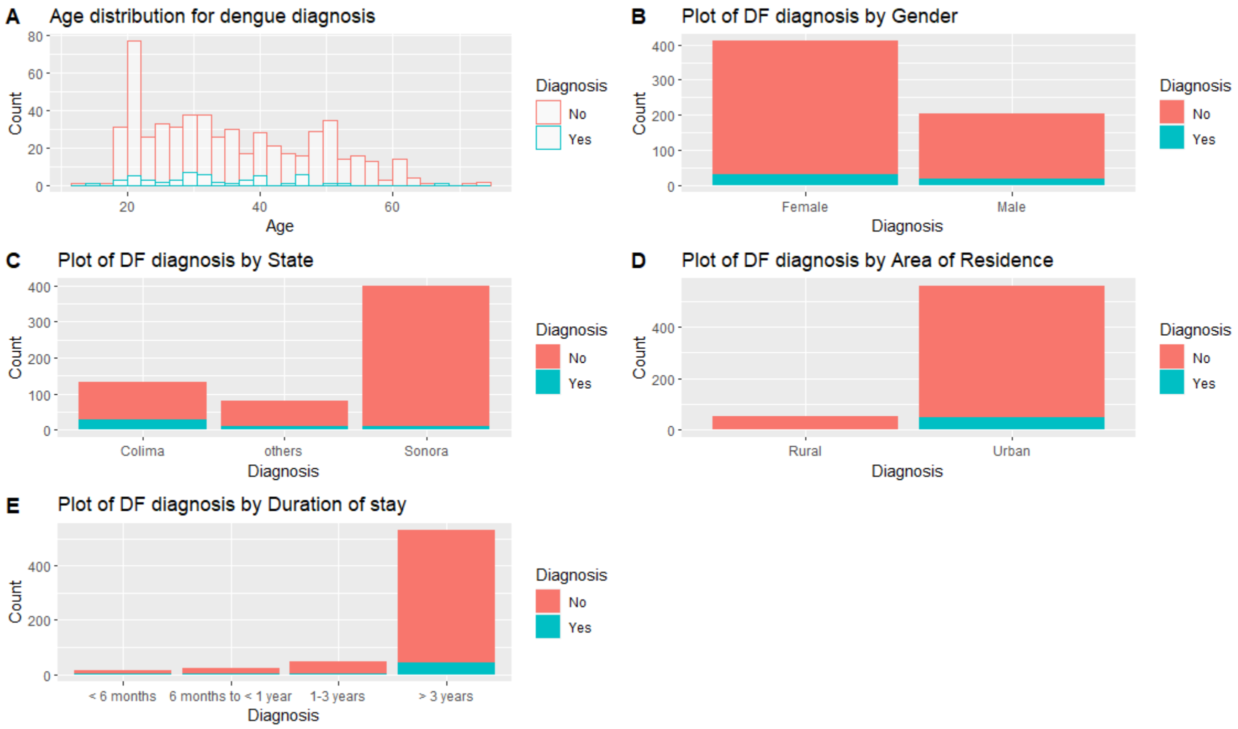

Figure S3. Distribution of dengue diagnosis by demographic factors.

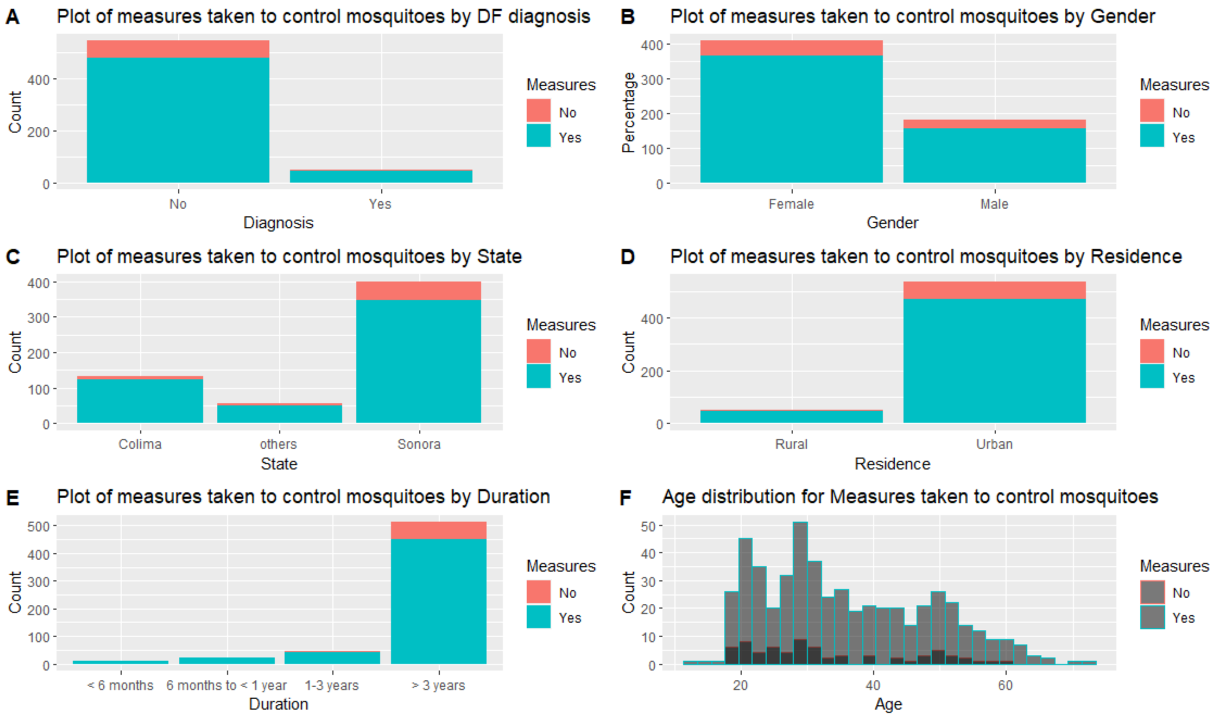

Figure S4. Distribution of mosquito control measures by demographic factors.
