# Supplementary material for: Determining Perceived Self-Efficacy for Preventing Dengue Fever in Two Climatically Diverse Mexican States: A Cross-Sectional Study
Source: Behav Sci (Basel). 2022 Mar 28;12(4):94. doi: 10.3390/bs12040094 (PMC9031455; doi:10.3390/bs12040094)
Supplement: Supplementary file 1 [file behavsci-12-00094-s001.zip › Treatment_health_seeking_behavior_dengue_5.18.21.pdf]

Treatment- and health-seeking behaviors for dengue fever

Informed Consent to Participate in a Research Study

Dr. Ubydul Haque, an Assistant Professor from the University of North Texas Health Science Center, is inviting you to participate in this study, which is approved by the North Texas Regional Institutional Review Board. The research survey will take no longer than 20 minutes.

Participation in this study will involve completing the below questionnaire which has six sections. The purpose of this study is to assess your treatment and health-seeking behavior for dengue fever. The results of this study may be published in scientific research journals or presented at professional conferences.

This survey is voluntary. All information collected through this survey is anonymous and will be stored in a password protected secure place. No participants will be identified. Please do not put your name, address, or any other personal identifiers on this survey. In the event you provide any identifying information on the survey, there is a potential risk of loss of confidentiality. The benefits of this study include helping scientists learn more about the factors that affect the knowledge of dengue fever and health-seeking behaviors towards prevention and treatment of dengue. The survey should be completed by the head of the household.

If you have any survey related questions/concerns, please contact Dr. Ubydul Haque at Mdubydul.Haque@unthsc.edu. If you have any questions about your rights or any other concerns, you may also contact the North Texas Regional Institutional Review Board on (817) 735-0409. Thank you!

Instructions: Please respond to the following questions by placing a mark ('X') in the answer box that corresponds to your response and/or fill in the blank where indicated. If multiple answer options are true, select all that apply.

Please confirm your voluntary participation and give your consent for data to be analyzed anonymously. *

☐ I confirm my voluntary participation & consent and confirm that I am at least 18 years of age

No ☐ **(Thank respondent and end survey)**

NOTE: You can skip any question or stop taking the survey at any time without any penalty or loss of benefits to which you are otherwise entitled.

Section 1: Socio-Demographics

1. What gender do you identify as?
- ☐ Male
- ☐ Female

2. Age____________________

3. What is your current employment status?
   ☐ Employed Full-Time
   ☐ Employed Part-Time
   ☐ Not employed, Seeking opportunities
   ☐ Not employed, Not seeking opportunities
   ☐ Retired
   ☐ Prefer not to say

4. What kind of area do you live in?
❑ Rural
❑ Suburban
❑ Urban

5. How long have you been living in this area?
☐ Below 6 months
☐ 6 months to < 1 year
☐ 1-3 years
☐ Above 3 years

6. What kind of home do you live in?
❑ Bungalow
❑ Single-family home/ landed properties
❑ Semi-Detached
❑ Terrace
❑ Townhouse
❑ Flat/Apartment
❑ Duplex
❑ Condominium
❑ Other (please specify) ____

7. How many individuals live in your household, including yourself?
❑ 1
❑ 2
❑ 3
❑ 4
❑ 5 or more

Section 2: Knowledge about Dengue Fever

8. How is dengue fever transmitted? ***(check all that apply)***

☐ Dengue is transmitted through infected mosquitos
☐ Dengue is transmitted via blood transfusion or organ transplantation

☐ Dengue is transmitted through contact with infected bodily fluids
☐ Dengue is transmitted from mother to fetus during pregnancy
☐ Other (please specify) ______________________________

9. How can the transmission of dengue fever via mosquitos be reduced? ***(check all that apply)***

☐ Using mosquito nets
☐ Having screens on windows/doors
☐ Insecticide spraying
☐ Covering open water containers
☐ Use of insect repellant
☐ Draining stagnant water
☐ Proper disposal of trash
☐ Other (please specify) ______________________________

10. What symptoms are associated with dengue fever? ***(check all that apply)***

☐ Fever
☐ Headache
☐ Joint Pain
☐ Muscle pin
☐ Pain behind the eyes
☐ Rash
☐ Nausea
☐ Vomiting
☐ Diarrhea
☐ Other (please specify) ______________________________

Section 3: Knowledge about Climate Change

11. Have you heard of climate change?
❑ Yes
❑ No
❑ Not sure

12. Do you think that climate change is currently occurring?
❑ Yes
❑ No
❑ Not sure

13. Do you think changes in climate can affect transmission of dengue fever?
❑ Yes
❑ No

- ❑ Not sure

If yes to question 13, answer question 14. Otherwise, skip to question 15.

14. What factors of climate change do you think affect diseases such as dengue fever ***(check all that apply)***?

- ☐ Excessive heat
- ☐ Excessive cold
- ☐ Changes in pattern of rainfall
- ☐ Frequent hurricanes
- ☐ Frequent flooding
- ☐ Not sure
- ☐ Other (please specify) ___________________

<u>Section 4: Attitudes about Climate Change and Dengue Fever</u>

15. There should be more education programs on mitigation strategies related to climate change.

- ❑ Strongly disagree
- ❑ Disagree
- ❑ Neither agree nor disagree
- ❑ Agree
- ❑ Strongly agree

16. There should be more education programs on early warning signs (e.g., flood/drought) related to climate change.

- ❑ Strongly disagree
- ❑ Disagree
- ❑ Neither agree nor disagree
- ❑ Agree
- ❑ Strongly agree

17. There should be increased knowledge of how climate change can affect dengue fever.

- ❑ Strongly disagree
- ❑ Disagree
- ❑ Neither agree nor disagree
- ❑ Agree
- ❑ Strongly agree

18. We can reduce the risk of dengue caused by climate change.

- ❑ Strongly disagree
- ❑ Disagree
- ❑ Neither agree nor disagree
- ❑ Agree

- ❑ Strongly agree

19. The government needs to take immediate action to mitigate dengue fever risk that may be because of climate change.
- ❑ Strongly disagree
- ❑ Disagree
- ❑ Neither agree nor disagree
- ❑ Agree
- ❑ Strongly agree

Section 5: Practices related to dengue fever

20. In the last year, did you take any measures to eliminate mosquitos in your household?
- ❑ Yes
- ❑ No
- ❑ Not sure

If yes to question 20, then answer questions 21-22. Otherwise, skip to question 23.

21. *What measures did you take for mosquito control?* (Please circle your answer)

Used insecticide sprays to reduce mosquitoes	Yes	No	Not Applicable
Cleaned water containers	Yes	No	Not Applicable
Covered water containers	Yes	No	Not Applicable
Treated water in water containers	Yes	No	Not Applicable
Used professional pest control to reduce mosquitoes	Yes	No	Not Applicable
Used screen windows to reduce mosquitoes	Yes	No	Not Applicable
Eliminated standing water around the house to reduce mosquitoes	Yes	No	Not Applicable
Used mosquito coils to reduce mosquitoes	Yes	No	Not Applicable
Cleaned garbage/trash	Yes	No	Not Applicable
Cut down vegetation around the house	Yes	No	Not Applicable
Used smoke to drive away mosquitoes	Yes	No	Not Applicable
Covered body with protective clothing	Yes	No	Not Applicable
Turned containers upside down to avoid water collection	Yes	No	Not Applicable
Disposed of old tires	Yes	No	Not Applicable

Treatment- and health-seeking behaviors for dengue fever

22. How often did you use measures to control mosquitos?
- ❑ Weekly
- ❑ Bi-weekly
- ❑ Every month
- ❑ Every few months
- ❑ Never

23. Are any of the following items present in your yard? (Please circle your answer)

Discarded tires	Yes	No	Not Applicable
Cans	Yes	No	Not Applicable
Plastic bottles	Yes	No	Not Applicable
Coconut shells	Yes	No	Not Applicable
Flower vases	Yes	No	Not Applicable
Holes in ground	Yes	No	Not Applicable
Water jars	Yes	No	Not Applicable
Water tanks	Yes	No	Not Applicable
Window/door screens	Yes	No	Not Applicable

24. At any time in the past 12 months, has anyone come into your dwelling to spray the interior walls against insects?
- ❑ Yes
- ❑ No

25. Do you use any kind of mosquito control **against larvae (baby mosquitos)** in your household?
- ❑ Yes
- ❑ No
- ❑ Not sure

26. Do you use any kind of mosquito control **against adult mosquitoes** in your household?
- ❑ Yes
- ❑ No

If yes to question 26, then proceed to next question. Otherwise, skip to question 29.

27. **What kind** of adult control do you use? (Select the appropriate box to indicate which kind of control you use)
    - ❑ **Mosquito nets**
    - ❑ **Household insecticide sprays**
    - ❑ **Fogging**

- ☐ **Mosquito swatter**
- ☐ **Repellent**
- ☐ **Other: ______________________________**

28. How often you use the respective chosen adult control in (27)?

	Daily	Weekly	Monthly	Every 3 months	> Every 3 months
**Mosquito nets**	☐	☐	☐	☐	☐
**Household insecticide sprays**	☐	☐	☐	☐	☐
**Fogging**	☐	☐	☐	☐	☐
**Mosquito swatter**	☐	☐	☐	☐	☐
**Repellent**	☐	☐	☐	☐	☐
**Other: ___________________**	☐	☐	☐	☐	☐

29. How often does your community engage in activities to clean the neighborhood?

☐ Weekly
☐ Biweekly
☐ Monthly
☐ Quarterly
☐ Every six months
☐ Once a while
☐ Other (please specify) _________________________

30. How often is waste disposal service available in your community?

☐ Weekly
☐ Biweekly
☐ Monthly
☐ Quarterly
☐ Every six months
☐ Once a while
☐ Absent
☐ Other (please specify) _________________________

31. How often is water supply service available in your community?

☐ Weekly
☐ Biweekly
☐ Monthly
☐ Quarterly
☐ Every six months
☐ Once a while
☐ Absent
☐ Other (please specify) _________________________

Section 6: Treatment-seeking approach and health seeking behavior

Treatment- and health-seeking behaviors for dengue fever

32. In the past one year, were you or anyone in your household diagnosed with dengue fever?
- ❑ Yes
- ❑ No
- ❑ Don't know

If Yes to question 32, proceed to next question. Otherwise, skip to question 34.

33. Where was dengue fever diagnosed?
- ☐ Healthcare center
- ☐ Self-diagnosis
- ☐ Other (please specify) ____

34. Are healthcare providers at nearby health centers available when needed?
- ❑ Yes
- ❑ No
- ❑ Don't know

35. In the past one year, has anyone in your neighborhood been diagnosed with dengue fever?
- ☐ Yes
- ☐ No
- ☐ Don't know

Section 7: Level of self-efficacy towards dengue prevention practices at the individual, household level and community level.

Using a scale of 0-10 where 0-not at all confident and 10-extremely confident, rate your confidence level to the following statements by circling the appropriate response.

36. I can change the water in plant pot trays every week.

**0**	**1**	**2**	**3**	**4**	**5**	**6**	**7**	**8**	**9**	**10**
**Not at all Confident**					**Moderately Confident**					**Extremely Confident**

37. I can clean the drain to prevent blockage every 7 days

**0**	**1**	**2**	**3**	**4**	**5**	**6**	**7**	**8**	**9**	**10**
**Not at all Confident**					**Moderately Confident**					**Extremely Confident**

38. I can always cover tightly all water containers inside and outside house

**0**	**1**	**2**	**3**	**4**	**5**	**6**	**7**	**8**	**9**	**10**
**Not at all Confident**					**Moderately Confident**					**Extremely Confident**

Treatment- and health-seeking behaviors for dengue fever

39. I can ensure that my children always put all garbage into closed bin

0 1 2 3 4 5 6 7 8 9 10

Not at all Moderately Extremely

Confident Confident Confident

40. I can go to see a doctor immediately when I become sick

0 1 2 3 4 5 6 7 8 9 10

Not at all Moderately Extremely

Confident Confident Confident

41. I can take my family member to see a doctor immediately they become sick

0 1 2 3 4 5 6 7 8 9 10

Not at all Moderately Extremely

Confident Confident Confident

42. I can change the water in the container under the fridge every week

0 1 2 3 4 5 6 7 8 9 10

Not at all Moderately Extremely

Confident Confident Confident

43. I can allow the health authority to fog and inspect my house at anytime

0 1 2 3 4 5 6 7 8 9 10

Not at all Moderately Extremely

Confident Confident Confident

44. I can work with my neighbors to do a weekly search and destroy any potential aedes breeding sites outside the house

0 1 2 3 4 5 6 7 8 9 10

Not at all Moderately Extremely

Confident Confident Confident

45. I can always put larvicides in water containers inside my house

Treatment- and health-seeking behaviors for dengue fever

0 1 2 3 4 5 6 7 8 9 10

Not at all Moderately Extremely

Confident Confident Confident

46. I can always ensure my family sleeps under a mosquito net every day/night

0 1 2 3 4 5 6 7 8 9 10

Not at all Moderately Extremely

Confident Confident Confident

47. How confident are you that you could talk to your neighbor about removing a container full of baby mosquitos behind his/her house?

0 1 2 3 4 5 6 7 8 9 10

Not at all Moderately Extremely

Confident Confident Confident

48. How confident are you that you could convince your neighbor to do 10 minutes weekly search and destroy any potential mosquito breeding sites?

0 1 2 3 4 5 6 7 8 9 10

Not at all Moderately Extremely

Confident Confident Confident

49. If your neighbor refused to destroy a container full of baby mosquitos behind his/her house, how confident are you that you can report him to the local Health Authority?

0 1 2 3 4 5 6 7 8 9 10

Not at all Moderately Extremely

Confident Confident Confident
